# Self-Glycolipids Modulate Dendritic Cells Changing the Cytokine Profiles of Committed Autoreactive T Cells

**DOI:** 10.1371/journal.pone.0052639

**Published:** 2012-12-20

**Authors:** Karsten Buschard, Jan-Eric Månsson, Bart O. Roep, Tatjana Nikolic

**Affiliations:** 1 Bartholin Instituttet, Rigshospitalet, Copenhagen, Denmark; 2 Institute of Neuroscience and Physiology, Sahlgrenska Academy, University of Gothenburg, Gothenburg, Sweden; 3 Department of Immunohematology and Blood Transfusion, Leiden University Medical Center, Leiden, The Netherlands; Istituto Superiore di Sanità, Italy

## Abstract

The impact of glycolipids of non-mammalian origin on autoimmune inflammation has become widely recognized. Here we report that the naturally occurring mammalian glycolipids, sulfatide and β-GalCer, affect the differentiation and the quality of antigen presentation by monocyte-derived dendritic cells (DCs). In response to sulfatide and β-GalCer, monocytes develop into immature DCs with higher expression of HLA-DR and CD86 but lower expression of CD80, CD40 and CD1a and lower production of IL-12 compared to non-modulated DCs. Self-glycolipid-modulated DCs responded to lipopolysaccharide (LPS) by changing phenotype but preserved low IL-12 production. Sulfatide, in particular, reduced the capacity of DCs to stimulate autoreactive Glutamic Acid Decarboxylase (GAD65) - specific T cell response and promoted IL-10 production by the GAD65-specific clone. Since sulfatide and β-GalCer induced toll-like receptor (TLR)-mediated signaling, we hypothesize that self-glycolipids deliver a (tolerogenic) polarizing signal to differentiating DCs, facilitating the maintenance of self-tolerance under proinflammatory conditions.

## Introduction

Sulfatide (3′-sulphogalactosylceramide) and its direct precursor galactosylceramide (β-GalCer) are glycolipids present in the nervous system, renal tubules and in islets of Langerhans [1]–[3]. Sulfatide and β-GalCer expressed in the islets of Langerhans are dominated by two variants, in which the fatty acid is fully saturated palmitic (C16) or lignoceric (C24) acid [4]. In pancreatic β-cells, sulfatide is located in the secretory granules where it is involved in the folding, trafficking and exocytosis of insulin [4], [5]. Glycolipids have been associated with autoimmunity since the treatment with α-GalCer isolated from marine sponges or with mammalian-derived sulfatide prevented spontaneous diabetes in non-obese diabetic (NOD) mice [6]–[8] and experimental autoimmune encephalomyelitis (EAE) [9], [10]. The presence of self-glycolipids in target tissues of autoimmune inflammation and the existence of sulfatide-specific autoantibodies in type 1 diabetes (T1D) [4], [11] or of sulfatide- and β-GalCer-specific autoreactive T cells in multiple sclerosis (MS) [12] underscores the potential relevance of these self-glycolipids in human autoimmune disease.

The discovery of a group of CD1 molecules being able to present glycolipid structures to T cells [13] has led to much attention to the role of CD1d-restricted presentation of glycolipids [14]–[18]. A function of self-glycolipids that extends beyond being antigens of the CD1-restricted immune response has been scarcely investigated. We have demonstrated that sulfatide inhibits activation of HLA-DR-restricted insulin-specific CD4 T cells [19] and that self-glycolipids alter cytokine response of a human monocytic line to lipopolysaccharide (LPS) [20]. Therefore, we hypothesized that sulfatide modulates the adaptive immune response through antigen presenting cells. Dendritic cells (DCs) are professional antigen presenting cells that maintain peripheral tolerance under steady state conditions [21], [22]. Immature DCs or monocytes are generally present in pancreatic islets where they are exposed to sulfatide or β-GalCer. Therefore, we investigated whether these self-glycolipids influence DC differentiation and their ability to activate autoreactive CD4 T cells.

## Materials and Methods

### Dendritic Cell Cultures

Dendritic cells (DCs) were generated as previously described [23]. Peripheral blood mononuclear cells were isolated from buffy-coats obtained from HLA-typed healthy blood donors. Monocytes were positively selected using CD14-magnetic beads and the MACS cell sorting system (Miltenyi Biotech, Bergisch Gladbach, Germany). Purified CD14+ monocytes (routinely >95% pure) were cultured for 6 days at 37°C, 5% CO_2_ in complete RPMI 1640 medium (Gibco Life Technologies, Breda, The Netherlands) supplemented with 8% FCS, 100 IU/ml penicillin and 100 IU/ml streptomycin, 800 U/ml rHu GM-CSF (Leucomax, Novartis Pharma, Arnhem, the Netherlands) and 1000 U/ml rHu IL-4 (Strathmann Biotech AG, Hanover, Germany). The medium was refreshed at day 3 of culture. β-GalCer or sulfatide were added directly to the cultures from a stock solution to obtain final concentrations of 30 nmol/ml. At day 6, DC maturation was induced by addition of 800 U/ml rHu GM-CSF and 100 ng/ml LPS (Shering-Plough, Uden, The Netherlands) and 1000 U/ml rHu IFNγ where appropriate.

For the phenotype analysis, FITC-labeled anti-human CD1a (clone HI-149), CD14 (clone M5E2), CD40 (clone 5C3), HLA-DR (clone G46-6), PE-labeled CD86 (clone IT2.2), CD80 (clone L307.4), CD83 (clone HB15e), CD54 (clone HA58) and isotype controls IgG1-PE and FITC (clone X40) were all obtained from BD Pharmingen (San Diego, CA). For each sample, 10000 live cells were analyzed on a FACSCalibur (Becton Dickinson) and analyses were performed using Flow Jo 7.5 software (TreeStar, Ashland, OR).

To induce cytokine release, DCs were cultured with irradiated CD40L-expressing fibroblasts and supernatants collected after 24 hrs [23]. The production of TNF and IL-12p70 by mature DCs was measured by ELISA CytoSets (Biosource, Camarillo, CA, USA). To evaluate changes in the cytokine production caused by the treatment with β-GalCer or sulfatide, data were normalized to the levels measured in untreated DC cultures.

For the analysis of TLR2- or TLR4-mediated signaling, DCs were generated as described above and supplemented with C24 sulfatide (30 nmol/ml) alone or combined with blocking anti-TLR2 (10 µg/ml) or anti-TLR4 (10 µg/ml) antibodies (eBioscienses, San Diego, CA, USA). At day 6, supernatants were collected from immature DC cultures and analyzed using cytokine array (Raybiotech inc., GA, USA).

### T Cell Proliferation and Cytokine Production

The polyclonal Glutamic Acid Decarboxylase (GAD65)-specific Th1 T cell line was established from a recent onset T1D patient as described earlier [24]. Clonal GAD65-specific T cells (clone PM1#11), expressing a Th0 cytokine profile, were cultured from PBMC derived from a Stiff-man syndrome patient before the onset of T1D [24], [25]. Expansion of the T cell response in the presence of GAD65 protein was used to measure the antigen uptake and processing dependent presentation. Experiments with the GAD339-352 peptide were designed to analyze the processing independent presentation. T cells and extensively washed HLA-matched DCs were cultured for 4 days in IMDM medium supplemented with 10% pooled human serum, 100 IU/ml penicillin and 100 IU/ml streptomycin. 0.5 µCi [^3^H]-thymidine was added during the final 18 hours of culture. [^3^H]-thymidine incorporation was determined by liquid scintillation counting. The results are presented as mean ± SD of triplicate cultures.

T cell cytokine release (IL-10, IL-13 and IFNγ) from PM1#11 was determined by ELISpot analysis following manufacturer’s guidelines. (U-CyTech, Utrecht, the Netherlands). The cytokine production was evaluated as described previously [23]. The results are shown as mean number of spots/10^4^ T cells ± SD of triplicate cultures.

### Glycolipid Preparation

Sulfatide and β-GalCer were isolated from porcine brain by extraction with chloroform/methanol/water (4∶8∶3; by vol.) and phase partition in chloroform/methanol (2∶1) as previously described [3]. Stock solutions (1 µmol/ml) were prepared by ultrasound sonification in RPMI 1640 medium without supplements before adding the desired amount to cell cultures. Prior to use, preparations of isolated glycolipids were tested for LPS contamination, which was routinely below detection level. A concentration of 30 nmol/ml of glycolipids has been used, unless stated otherwise.

### Toll Like Receptor 2 and 4 Signaling

HEK 293 cells transfected with TLR2 or TLR4 and IL-8-reporter gene constructs were used as previously described [26]. Sulfatide and β-GalCer were added to HEK 293 cells in the range of 3 to 12 nmol/ml and IL-8 production was measured in supernatants after 24 hours using a commercial kit (Sanquin, Amsterdam, The Netherlands), by following the manufacturer’s recommendations.

### Statistical Analysis

The Mann-Whitney *U* test was used to compare results obtained with variously treated DCs. Comparison of the changes in cytokine production by the GAD65-specific clone was performed using 2-way ANOVA with Bonferroni correction. Significance was defined at the p<0.01, unless stated differently.

## Results

### Sulfatide and β-GalCer Interfere with DC Differentiation

**Figure 1:**
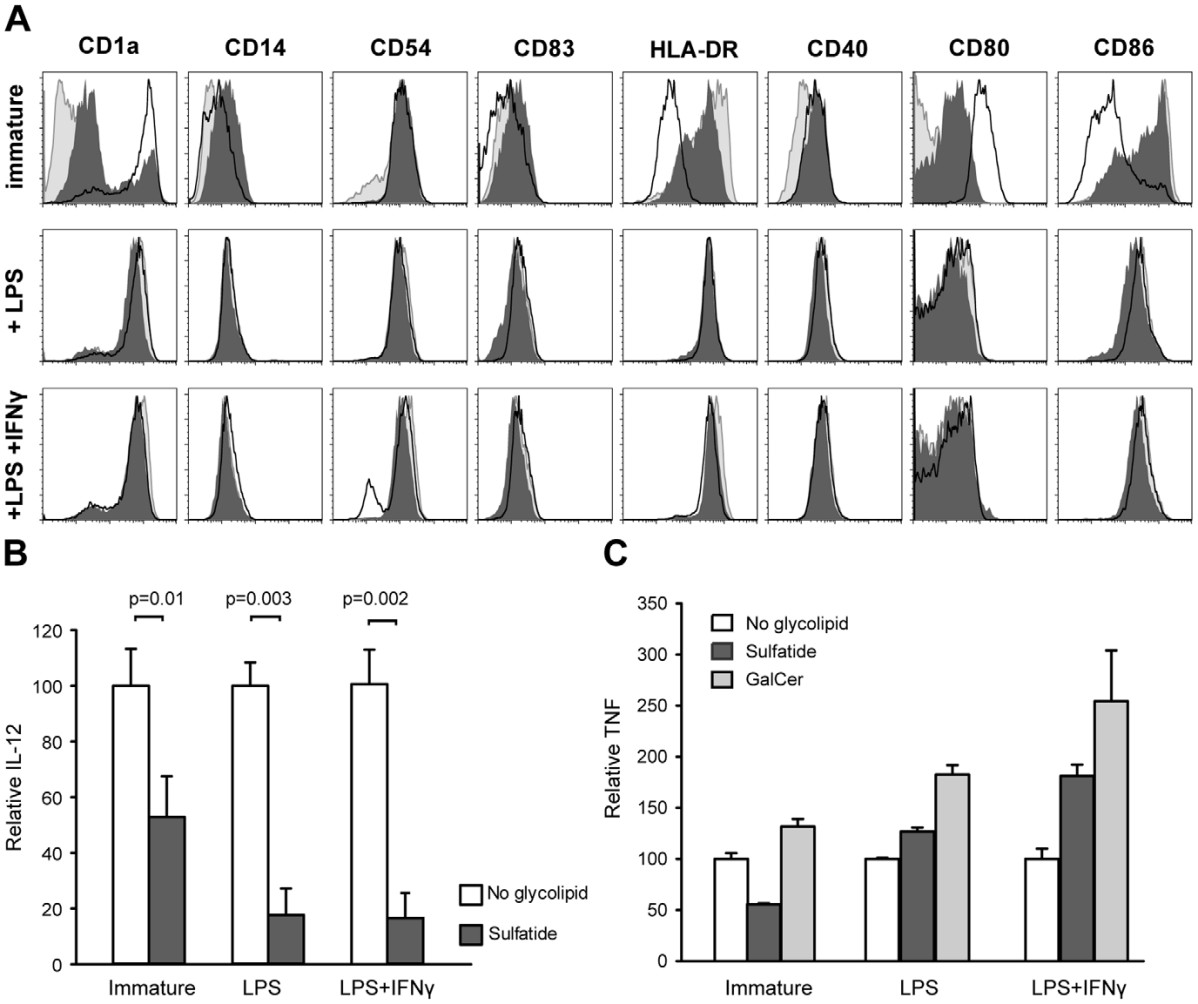
Sulfatide and β-GalCer modify the phenotype of immature DCs. (A) The phenotype analysis of immature dendritic cells (top row) and after maturation with LPS (center row) or LPS+IFNγ (bottom row). Dendritic cells were differentiated from monocytes with GM-CSF and IL-4 alone (empty histograms) or in the presence of sulfatide (dark grey histograms) or β-GalCer (light grey histograms). After 6 days (immature DCs) or 8 days (mature DCs - LPS or LPS+IFNγ), cells were harvested and stained with fluorescent antibodies. Histograms are representative of three independent experiments. Stimulated production of IL-12p70 (B) and TNF (C) were measured in the supernatant collected at the end of each culture condition as detailed under A. The cytokine concentration was determined by ELISA. Graphs show relative cytokine expression normalized to the corresponding cytokine levels measured in unstimulated cultures from two experiments (n = 2).

Dendritic cells were generated from CD14+ monocytes by cultivation for 6 days in presence of GM-CSF and IL-4 (untreated DCs). Sulfatide or β-GalCer was added from the beginning of culture and the resulting DC phenotype was analyzed by flow cytometry (Figure 1). Compared to untreated DCs, sulfatide- or β-GalCer-treated immature DCs showed lower expression of CD1a and CD80 but higher expression of HLA-DR and CD86. These phenotype differences disappeared upon maturation of DCs with LPS or LPS+IFNγ (Figure 1). The changes of surface expression of co-stimulatory molecules CD86, CD80 and HLA-DR induced by glycolipids were restricted to early exposure to sulfatide, i.e. from the beginning of the *in vitro* differentiation process. If glycolipids were added during LPS-induced maturation, we observed no phenotypic differences between untreated and sulfatide- or β-GalCer-treated DCs (data not shown). Untreated DCs matured with LPS or LPS+IFNγ produce high IL-12p70 and TNF and we assessed the consequences of sulfatide- or β-GalCer-treatment relative to the untreated cultures (Figure 1). Sulfatide treatment significantly reduced the capacity of DCs to produce IL-12p70 both before and after maturation with LPS or LPS+IFNγ (p<0.01, Mann-Whitney). Sulfatide also reduced the capacity of immature DCs to produce TNF. In contrast, sulfatide- or β-GalCer- treated DCs increased the TNF production after maturation with LPS or LPS+IFNγ compared to untreated mature DC. The effect of sulfatide on DCs was due to the C24:0 isoform and not the C16:0, which displayed no stimulation (data not shown).

### Self-glycolipid Treated DC Modulate Cytokine Production by Autoreactive T Cells

**Figure 2:**
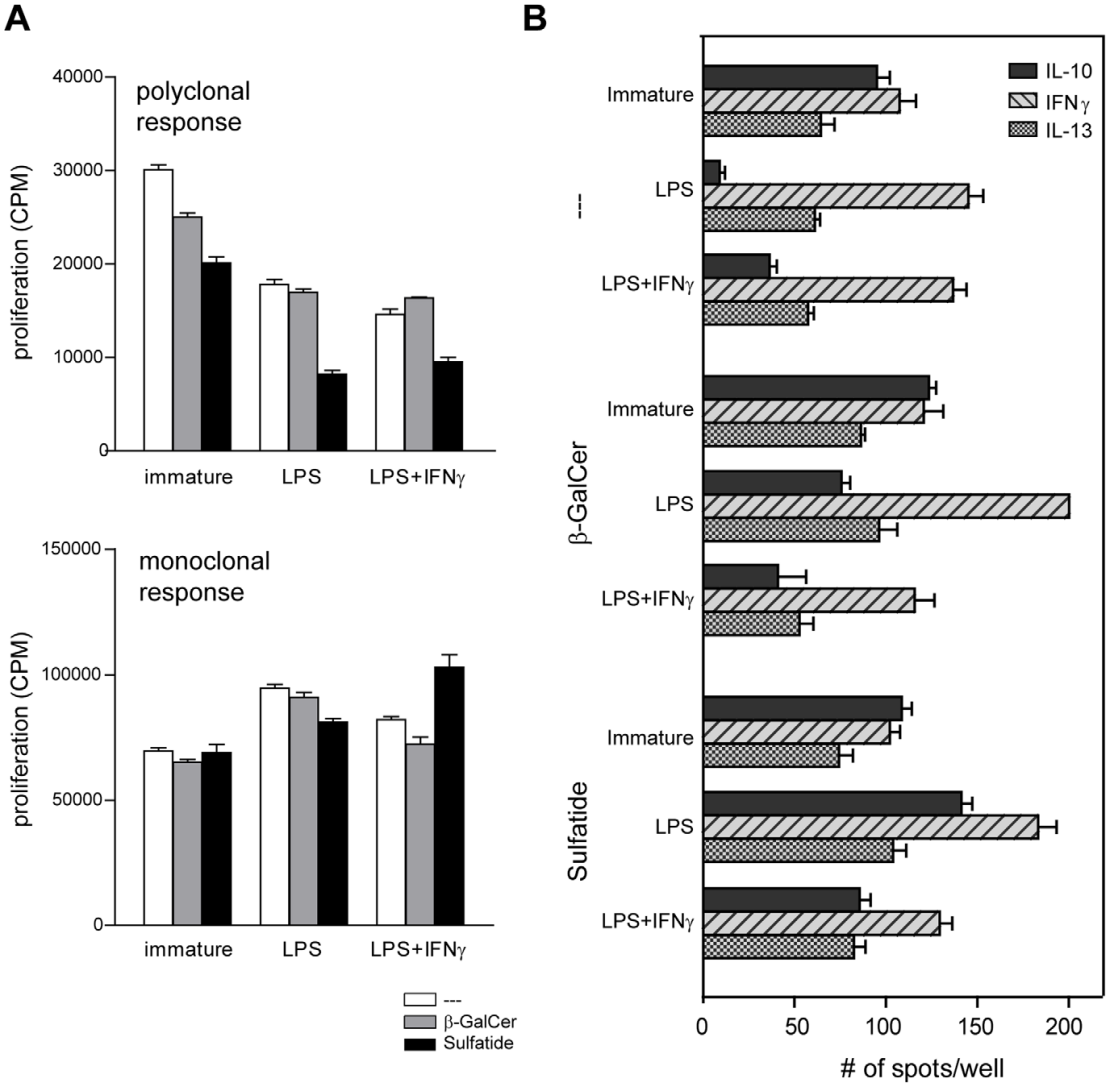
Sulfatide reduces the capacity of DCs to induce proliferation of GAD65-specific Th1 cells and diverts LPS-induced inhibition of IL-10 production by the GAD65-specific clone. (A) GAD65-specific cells were incubated for 4 days with GAD65 protein-pulsed immature or LPS or LPS+IFNγ-matured DCs which were not exposed to self-glycolipids (white bars), modulated with β-GalCer (grey bars) or with sulfatide (black bars). 3H thymidine was added during the last 18hrs of the culture. Graphs show proliferation of GAD65-specific cells (polyclonal response – Th1 T cell line; monoclonal response – PM1#11 clone), measured by 3H thymidine incorporation. The results are presented as mean counts per minute (CPM) ± SD of triplicate cultures. (B) The cytokine production by the GAD65-specific T cell clone (PM1#11) as measured by ELISpot analysis. T cells were incubated with DCs (immature, LPS-matured or LPS+IFNγ-matured) pulsed with GAD65 protein, which were untreated (–-) or modulated with β-GalCer or Sulfatide. Dark grey bars depict IL-10, light hatched bars depict IFNγ and square-hatched bars depict IL-13 production. The data show mean number of spots/10^4^ T cells ± SD of triplicate cultures. Three independent experiments were performed (n = 3).

The capacity of self-glycolipid-treated DCs to process and present the GAD65-protein was tested by analyzing the stimulation of GAD65-specific T cells. Immature, LPS-, or LPS+IFNγ-matured sulfatide-treated DCs induced lower proliferation of polyclonal GAD65 T cells compared to β-GalCer- or untreated DCs (Figure 2). Correspondingly, polyclonal GAD65 specific T cells produced lower amounts of IFNγ when stimulated with sulfatide-treated DCs (data not shown).

To assess whether self-glycolipids modulate stimulatory capacity of DCs beyond antigen processing, we analyzed the effects of self-glycolipid treated DC on clonal GAD65-specific T cells. Sulfatide-treated DCs were equally potent as β-GalCer and untreated DCs to induce high T cell proliferative responses to either GAD65 protein or a specific peptide epitope thereof (GAD339-352) (Figure 2 and not shown). Next, we analyzed the cytokine production by the GAD65-specific clone stimulated with β-GalCer or sulfatide-treated DCs. We chose this particular T cell clone because of its Th0 cytokine profile (similar production of IFNγ, IL-10 and IL-13), which was shown to be subject to modification when stimulated by immunomodulatory 1,25VitD_3_-treated DCs [23]. The GAD65-specific clone did not change the Th0 cytokine profile when stimulated with immature β-GalCer or sulfatide-treated DCs, compared to immature untreated DCs (Figure 2). The maturation of untreated DCs significantly suppressed IL-10 production by the GAD65 clone increasing the IFNγ:IL-10 ratio from 1.1 with untreated immature DCs to 17.5 with untreated LPS-matured DCs (p<0.0001, 2-way ANOVA), reflecting the Th1 polarization as described earlier [27]. The β-GalCer or sulfatide-treated LPS-matured DC stimulated high IFNγ production by the GAD65 clone but retained significant IL-10 production, compared to the untreated LPS-matured DCs (Figure 2). As a result, the IFNγ:IL-10 ratio was reversed from 17.5 with untreated LPS-matured DCs to 2.9 with β-GalCer-treated and to 1.3 with sulfatide-treated LPS-matured DCs. Similar but less pronounced effect on IL-10 production was observed with β-GalCer-treated and sulfatide treated DCs matured with LPS+IFNγ. The production of IL-13 by the GAD65 clone was not significantly affected by either maturation or self-glycolipid treatment (Figure 2). In conclusion, the treatment of DCs with self-glycolipids significantly reduced the capacity of mature DCs to suppress IL-10 production and induce a Th1 polarization of autoreactive T cells (p<0.0001, 2-way ANOVA).

### Sulfatide and β-GalCer Signal through TLR2 and TLR4

**Figure 3:**
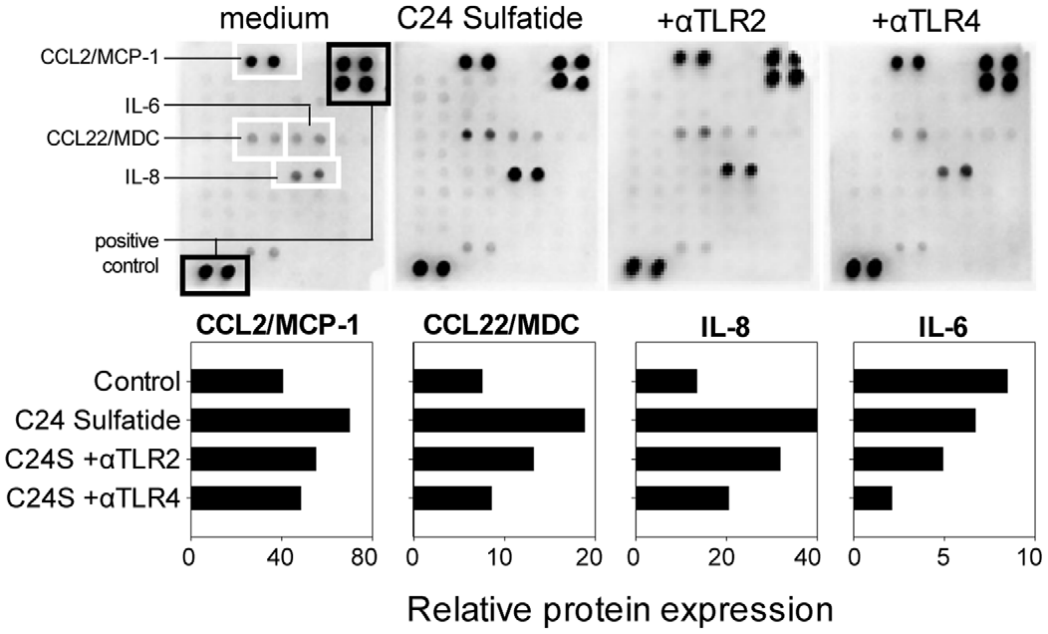
TLR2 and TLR4 are involved in sulfatide signaling in DCs. CD14+ monocytes were cultured for 6 days in the presence of GM-CSF and IL-4 to obtain immature DCs. From the beginning of culture, 10 µg/ml of mouse anti-human TLR2 or TLR4 were added and allowed to incubate for 1 hour before addition of sulfatide (C24S). Supernatants were collected on day 6 of the culture and chemokines/cytokines were measured using a cytokine array. All signals were normalized to the positive controls and represented as horizontal bars. Both anti-TLR2 and anti-TLR4 were able to reduce the release of chemokines and cytokines induced by sulfatide. The data show cumulative values from two independent DC cultures.

Since we previously demonstrated that self-glycolipids signal through CD14 [20], we analyzed whether TLR-mediated signaling plays a role in the functional modulation displayed by sulfatide-exposed DCs. Since of the two forms of sulfatide that are naturally produced in the islets of Langerhans, sulfatide isoform C16:0 blocks TLR4 signaling (20), we used the isoform C24:0 in following experiments. Supernatants from immature DCs generated in the presence of sulfatide C24:0 contained increased amounts of chemokines produced by dendritic cells CCL2, CCL22 and IL-8 in comparison with untreated immature DCs (Figure 3). The chemokine production induced by C24 sulfatide was reduced when anti-TLR2 and anti-TLR4 blocking antibodies were added to the culture.

**Figure 4:**
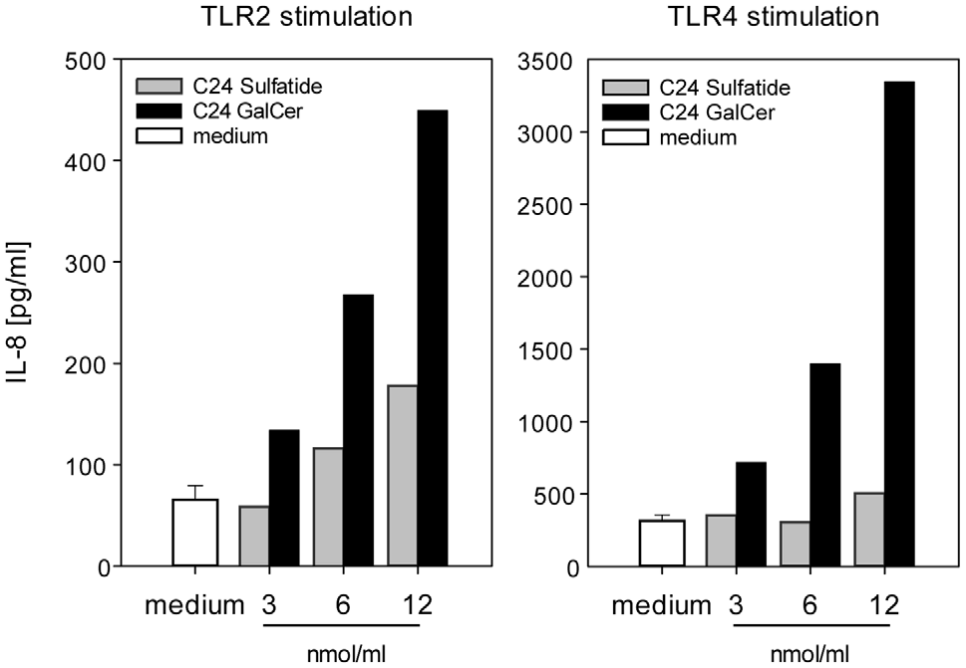
Sulfatide and β-GalCer induce TLR2 and TLR4-mediated signaling. HEK 293 cells transfected with constructs containing TLR2 or TLR4, and IL-8 as a reporter gene under NFkB promoter control. Sulfatide or β-GalCer isoform C24 were added to HEK 293 transfectants in the range of 3–12 nmol/ml. Supernatants were measured for the presence of IL-8, which is released upon TLR activation. Both sulfatide (grey bars) and β-GalCer (black bars) were able to engage TLR2 and TLR4 with β-GalCer being a particularly potent TLR4 agonist. The data show a representative experiment.

Functional recognition of C24 sulfatide by TLR2 and TLR4 was substantiated by testing sulfatide and β-GalCer on HEK293 cells transfected with TLR2 or TLR4 and a reporter gene IL-8 controlled by NFkB promoter, as previously described [26]. C24 sulfatide was able to induce IL-8 production through TLR-2 and β-GalCer was able to induce signaling through both TLR2 and TLR4 in a dose dependent manner (Figure 4).

## Discussion

Dendritic cells are antigen presenting cells that bridge innate and acquired immunity. In a non-inflammatory environment, DCs maintain peripheral tolerance to self-antigens [21], [22]. Autoimmune diseases such as type 1 diabetes or MS are a consequence of a faulty self-tolerance allowing autoreactive T cells to emerge and subsequently destroy target tissues. We report how naturally occurring glycolipids synthesized in pancreatic islets of Langerhans interfere with acquired immunity through affecting the phenotypic and functional features of DCs, which subsequently deviate committed autoreactive T cells from a Th1 towards IL-10-producing profile even under extreme Th1 polarizing conditions. TLR2 and TLR4 are involved in self-glycolipids signaling.

Since the discovery that lipid antigens can be presented by the non classical CD1 system [8], CD1d presentation of glycolipids has gained a lot of attention and several studies have described that lipid antigens of non mammalian origin like α-GalCer from sponges can prevent or reduce autoimmune diseases through either boosting NKT cells or the induction of Th2 deviation of the immune response [6]–[10]. Glycolipids have been primarily investigated as antigens of the innate immune system. This is the first study demonstrating a role of sulfatide as direct modulator of the adaptive immune response through dendritic cells. Sulfatide may affect insulin processing by interfering with intracellular phagosome-lysosome fusion leading to reduced insulin presentation by professional antigen presenting cells [19]. This may explain why sulfatide inhibited the proliferation of insulin-specific autoreactive T cells in response to insulin protein while sulfatide had no effect on insulin B (11–27) peptide or anti-CD3 induced polyclonal T cell proliferation [19]. Our current data also show that sulfatide treatment of DCs inhibited proliferation of polyclonal responses to naturally processed GAD65 protein, without affecting proliferative responses to peptide epitopes of GAD65. Importantly, sulfatide treatment of DCs modified the cytokine production of the GAD65-specific T cell clone by diverting the strong Th1 response induced by LPS (and IFNγ) into IL-10 dominating T cell response. Thus, apart from direct presentation to NKT cells via the CD1 system, self-glycolipids may also act as an organ-specific tolerance-inducing substance modulating local DCs to provide a tolerogenic signal and impair cytokine secretion by infiltrating autoimmune T cells.

Self-glycolipids trigger Toll-like receptors (TLR) on DCs and may thus regulate immune tolerance in the pancreas. Phosphatidylserine (PS) from helminths interacts with TLR2 causing a polarization of immature DCs into Th2-inducers, which subsequently activate regulatory T cells that produce IL-10 [26]. We also found that self-glycolipids containing lignoceric acid (C24:0) signal through TLR2 and TLR4 but in contrast to lipid antigens from helminths, self-glycolipids predominantly modulated differentiation of monocytes into immature DCs and did not make DCs refractory to maturation signals. Modulation with sulfatide did imprint DCs with a stable low IL-12 production capacity, a key proinflammatory cytokine promoting Th1 cells. Modulated DCs also produced more TNF, which can support induction of antigen-specific regulatory T cells [28]. Considering the importance of regulatory T cells in postponing overt diabetes during ongoing insulitis [29] and the TLR family as key regulators of both innate and adaptive immune responses [30], our findings merit further investigation into sulfatide activated signaling pathways.

We discovered that of the two predominant variants of sulfatide that are expressed in islets of Langerhans, sulfatide containing lignoceric acid (C24:0) was able to induce partial, impaired maturation of DCs, as illustrated by changes in phenotype and cytokine production. The tissue specificity of this sulfatide variant supports a relevance of our findings to the pathogenesis of type 1 diabetes, which is the classic result of selective autoimmune T cell-mediated destruction of insulin-producing β-cells [31]. Indeed, a recent study reported that the prevention of diabetes in NOD mice by sulfatide is due to the C24:0 isoform and not the C16:0 sulfatide [32].

If local presentation of sulfatide is central to maintaining a proper immune balance in the islets of Langerhans, the possible consequences of reduced levels or even completely absent production of sulfatide may be crucial to human disease. Sulfatide and β-GalCer differentially regulate cytokine production by human mononuclear cells and β-cells [20], [33], [34], and a disturbed balance between these two molecules could contribute to an increased β-cell vulnerability to destructive cytokines. Alterations in sulfatide and β-GalCer levels may be affected upon metabolic stress in β-cells when the amount of sulfatide is significantly reduced [35] or by islet- infiltrating NK cells, which express arylsulphatase, which converts sulfatide into β-GalCer [36]. In humans at risk of developing type 1 diabetes, β-cell dysfunction is characterized by hyper-production of insulin [37]. Insulin is a key autoantigen in type 1 diabetes, giving rise to both anti-insulin autoantibodies and T cell autoreactivity in preclinical models and new-onset patients [38], [39]. Sulfatide protects insulin-producing cells from cytokine-induced apoptosis [40] and increased insulin production together with reduced sulfatide expression may be an unfortunate combination negatively affecting local immune homeostasis and, hence, may serve as an important checkpoint in the pathogenesis of type 1 diabetes.
